# Exploring potential biomarkers for acute myocardial infarction by combining circadian rhythm gene expression and immune cell infiltration

**DOI:** 10.1038/s41598-025-88568-2

**Published:** 2025-02-01

**Authors:** Xiao Yu, Xiaopeng Zhang, Hazrat Bilal, Chang Shi, Lei Sun

**Affiliations:** 1 Department of Pathology and Forensic Medicine, College of Basic Medical Sciences, Dalian Medical University, Dalian, China; 2 Department of Pathology, First Affiliated Hospital, Dalian, Liaoning Province China

**Keywords:** Circadian rhythm-related genes, Acute myocardial infarction, Biomarker, Immune infiltration, Bioinformatics analysis, Cardiology, Cardiovascular biology

## Abstract

Current diagnostic biomarkers for acute myocardial infarction (AMI), such as troponins, often lack specificity, leading to false positives under non-cardiac conditions. Recent studies have implicated circadian rhythm and immune infiltration in the pathogenesis of AMI. This study hypothesizes that analyzing the interplay between circadian rhythm-related gene expression and immune infiltration identify highly specific diagnostic biomarkers for AMI. Our results demonstrated differential expression of 15 circadian rhythm-related genes (CRGs) between AMI patients and healthy individuals, with five key genes—JUN, NAMPT, S100A8, SERPINA1, and VCAN identified as key contributors to this process. Functional enrichment analyses suggest these genes significantly influence cytokine and chemokine production in immune responses. Immune infiltration assessments using ssGSEA indicated elevated levels of neutrophils, macrophages, and eosinophils in AMI patients. Additionally, we identified potential therapeutic implications with 13 pivotal miRNAs and 10 candidate drugs targeting these genes. The Benjamini–Hochberg method was employed to adjust for multiple testing, and the results retained statistical significance. RT-qPCR analysis further confirmed the upregulation of these five genes under hypoxic conditions, compared to controls. Collectively, our findings highlight the critical role of CRGs in AMI, providing a foundation for improved diagnostic approaches and novel therapeutic targets.

## Introduction

Myocardial infarction (MI) remains a leading global health challenge, significantly contributing to mortality and long-term disability worldwide^1^. Acute myocardial infarction (AMI), often triggered by sudden coronary events, can lead to severe outcomes, including myocardial rupture and sudden cardiac death, particularly when medical intervention is delayed^2^. The initiation of precise diagnosis and prompt therapeutic measures are of paramount importance for AMI patient prognosis. Thus, the prompt detection of AMI at the initial signs of chest pain is vital for averting detrimental consequences and enhancing patient survival rates. Extensive research has highlighted the utility of various biomarkers in diagnosing AMI, including myoglobin, cardiac troponin I (cTnI), and creatine kinase-MB (CK-MB)^3^. Among these, myoglobin serves as the earliest indicator, detectable within three hours of the onset of chest pain. However, it is predominantly indicative of skeletal muscle injury rather than myocardial damage^4,5^. Conversely, the cTnI and CK-MB, while comprehensive, tend to manifest later and therefore do not support the most timely diagnosis of AMI^6,7^. Moreover, in addition to AMI, elevations in cTnI could occur in a number of conditions, diminishing its specificity for diagnosis^8^. This often leads to missed opportunities for optimal treatment. Consequently, there is a critical need for novel biomarkers that can more precisely and reliably diagnose AMI.

Circadian rhythms, endogenous biological cycles inherent to most living organisms, orchestrate a wide range of physiological, mental, and behavioral processes. This regulation is achieved through the tightly coordinated modulation of gene expression and biochemical functions. Key genes involved in circadian rhythms include CLOCK (Clock Circadian Regulator), BMAL1 (Basic Helix-Loop-Helix ARNT Like 1), PER1/2/3 (Period Circadian Regulator1/2/3), CRY1/2 (Cryptochrome Circadian Regulator1/2), and others such as TIM (Timeless Circadian Regulator), NR1D1 (Nuclear Receptor Subfamily 1 Group D Member 1), NR1D2 (Nuclear Receptor Subfamily 1 Group D Member 2), CSNK1D (Casein Kinase 1 Delta), and CSNK1E (Casein Kinase 1 Epsilon)^9,10^. Under physiological conditions, circadian rhythm-related genes (CRGs) are involved in regulating heart rate, cardiac electrophysiology, blood pressure, blood coagulability, and vascular tone^11,12^. Emerging evidence suggests that, in addition to regulating cardiovascular physiologic processes, circadian rhythms also influence cardiovascular diseases, including atherosclerosis and thrombosis and myocardial injury subsequent to MI^13–15^. These findings illuminate the potential of CRGs to enhance the early diagnosis of AMI, offering promising avenues for therapeutic intervention and improved patient outcomes.

Recent studies have increasingly underscored the critical impact of immune cell infiltration on the development and progression of AMI. This process is involved in various stages of coronary artery atherosclerosis, such as lipid core enlargement, fibrous cap degradation, and plaque angiogenesis, which collectively elevate the risk of plaque rupture and thrombosis^2,16^. Furthermore, immune infiltration plays a vital role in the course of AMI by mediating injury and repair mechanisms^17,18^. Despite the acknowledged importance of immune cells in these processes, significant gaps remain in our understanding of their specific roles post-MI, particularly concerning inflammatory cell functions. Such studies are essential not only for elucidating the mechanisms at play but also for exploring the potential of immune cell profiles as early diagnostic markers for AMI.

Extant literature suggests that CRGs significantly influence inflammatory responses in heart failure^19,20^. This insight underpins our hypothesis that a combined analysis of CRGs and immune infiltration could enhance the precision in identifying diagnostic biomarkers for AMI. In our study, we performed a systematic evaluation of the expression of CRGs and their correlation with immune infiltration in AMI patients. This study aims to investigate the interaction between CRGs expression and immune infiltration in AMI, with the objective of identifying more precise diagnostic biomarkers and uncovering potential therapeutic targets.

## Materials and methods

### Collection of datasets

Gene expression profiles GSE48060 and GSE66360 were retrieved from the Gene Expression Omnibus (GEO). Both datasets were produced using the GPL570-Affymetrix Human Genome U133 Plus 2.0 Array [HG-U133_Plus_2]. Specifically, GSE48060 includes peripheral blood samples from 31 patients with acute myocardial infarction (AMI) and 21 control individuals with normal cardiac function. The GSE66360 dataset comprises peripheral blood samples from 49 AMI patients and 50 healthy controls.

### Data preparation and identification of CRDEGs

Raw data from the datasets were converted into expression matrices using the “limma” package in R (Version 4.2.1)^21^. The batch effects were removed using the “sva” package after merging the two datasets (GSE48060, GSE66360)^22^. Differential expression genes (DEGs) were determined using the “limma” package, with a significance threshold set at |log2FC|> 1 and *p* < 0.05. Results were displayed using volcano plots and heatmaps created with the “ggplot2” and “pheatmap” packages, respectively. Circadian rhythm-related genes (CRGs) were derived from prior studies and intersected with DEGs to identify circadian rhythm-dependent DEGs (CRDEGs), which were visualized using “ggplot2”^23,24^. CRGs were obtained via intersecting the DEGs from each array and the circadian rhythm-related genes using a Venn Diagram, and they were visualized as a Heatmap with R package “ggplot2”. The overlapped CRDEGs among the two arrays were eventually obtained.

### Functional enrichment analysis

Subsequently, functional enrichment analysis of differential CRDEGs, including Gene Ontology (GO) and Kyoto Encyclopedia of Genes and Genomes (KEGG), were performed using the “clusterProfiler” R package^25–28^. The pathways with *p* < 0.05 were identified as significant.

### PPI network construction and hub CRDEGs identification

The PPI network of the differential CRDEGs was constructed via the STRING database (https://www.string-db.org/) with the cutoff interaction score set at 0.7. Then the top 10 hub CRDEGs with the highest maximal clique centrality (MCC, Bottlenecks, Degree, DMNC, and EPC) values were selected via the “Cytohubba” plugin of Cytoscape and visualized using Cytoscape software (version 3.10.1).

### Infiltration analysis of immune cells and functions

The infiltrating score of 24 immune cells in healthy and AMI groups were calculated with single-sample gene set enrichment analysis (ssGSEA) via the “gsva” R package^29^ and visualized by heatmap using the “pheatmap” package^30^. The box plots were used to compare and visualize the ssGSEA scores of infiltrated immune cells between the healthy and AMI samples by “ggpubr” R package^31^. The correlation heatmap, which revealed the correlation of 24 types of immune cells and related functions, was performed using the “corrplot” R package.

### Construction and verification of the diagnostic model

To screen the CRDEGs with diagnostic potential, we analyzed the relationship among 5 CRDEGs (JUN, NAMPT, S100A8, SERPINA1, and VCAN) with immune cells using Spearman’s correlation analysis via the “ggcorrplot” R package for predicting AMI^32,33^. Then the 5 CRDEGs were subjected to Logistic regression analysis to construct a nomogram model via the “rms” R package. To evaluate the diagnostic performance of feature genes, the ROC was plotted using the “pROC” R package.

### Identification of pivotal miRNAs and candidate drugs

The candidate drugs were determined using the DSigDB database. The access of the Targetscan database and DSigDB database are acquired through Enrichr (http://amp.pharm.mssm.edu/Enrichr/) platform.

### Cell culture and processing of cardiomyocyte cell line

Human AC16 cardiomyocytes (Wuhan sunncell Biotech Co.,Ltd) were cultured in Dulbecco’s Modified Eagle’s Medium (DMEM, Gibco) supplemented with 10% FBS (Yeasen) and 1% penicillin–streptomycin (Cytiva) at 37℃ in an incubator containing 5% CO_2_. A hypoxia incubator chamber (STEMCELL Technologies, Canada) connected to a Proox Model 21 controller (BioSpherix, Redfield, NY) was used to establish a hypoxic environment. Thereafter, the hypoxic conditions were built through 24 h exposure of cells to the hypoxic environment (94% N_2_, 1% O_2_, and 5% CO_2_).

### Quantitative real-time PCR

Total RNA isolation was carried out with using TRizol reagent according to the manufacturer’s instructions (Sevenbio, SM139). All-in-one First Strand cDNA Synthesis Kit III (Sevenbio, SM135) was used to reverse transcribe cDNA. The reaction mixtures containing SYBR Green (Sevenbio, SM143) were composed following the manufacturer’s protocol and then CT values were obtained using a qPCR platform (Bioer, LineGene9600 FQD-96a v1.0.13 RC 20,200,911, Hangzhou, China). The genes expression levels of JUN, NAMPT, S100A8, SERPINA1, and VCAN in AC16 were measured using RT-qPCR. The ACTB gene served as the reference gene for the data. Relative quantitation was performed using the 2^−△△CT^ method. Primer details are shown in Table 1.

**Table 1:** Sequences of the primers used for RT-qPCR.

Name (homo)	Primers for RT-qPCR (5′-3′)
ACTB	Forward: GGGAAATCGTGCGTGACATT Reverse: GGAACCGCTCATTGCCAAT
S100A8	Forward: ATGCCGTCTACAGGGATGAC Reverse: ACTGAGGACACTCGGTCTCTA
SERPINA1	Forward: GGAGGCTCAGATCCATGAAGG Reverse: GGTGTCCCCGAAGTTGACAG
VCAN	Forward: GTAACCCATGCGCTACATAAAGT Reverse: GGCAAAGTAGGCATCGTTGAAA
NAMPT	Forward: ATCCTGTTCCAGGCTATTCTGT Reverse: CCCCATATTTTCTCACACGCAT
JUN	Forward: TCCAAGTGCCGAAAAAGGAAG Reverse: CGAGTTCTGAGCTTTCAAGGT

### Statistical analysis

Statistical analyses were performed using R software (version 4.2.1), SPSS Statistics (version 26.0) and GraphPad Prism (version 10.1.2). Continuous variables were expressed as mean ± SD or median (quartile range). The Student’s t-test was employed to analyze continuous variables with normal distribution. Categorical variables were presented as numbers (percentages) and analyzed using the chi-square test. The Benjamini–Hochberg procedure was used to correct for multiple comparisons. Statistical significance was set at (*p < 0.05, **p < 0.01, ***p < 0.001, ****p < 0.0001, ns: not significant).

## Results

### Differential CRGs between AMI patients and healthy controls

**Fig. 1:**
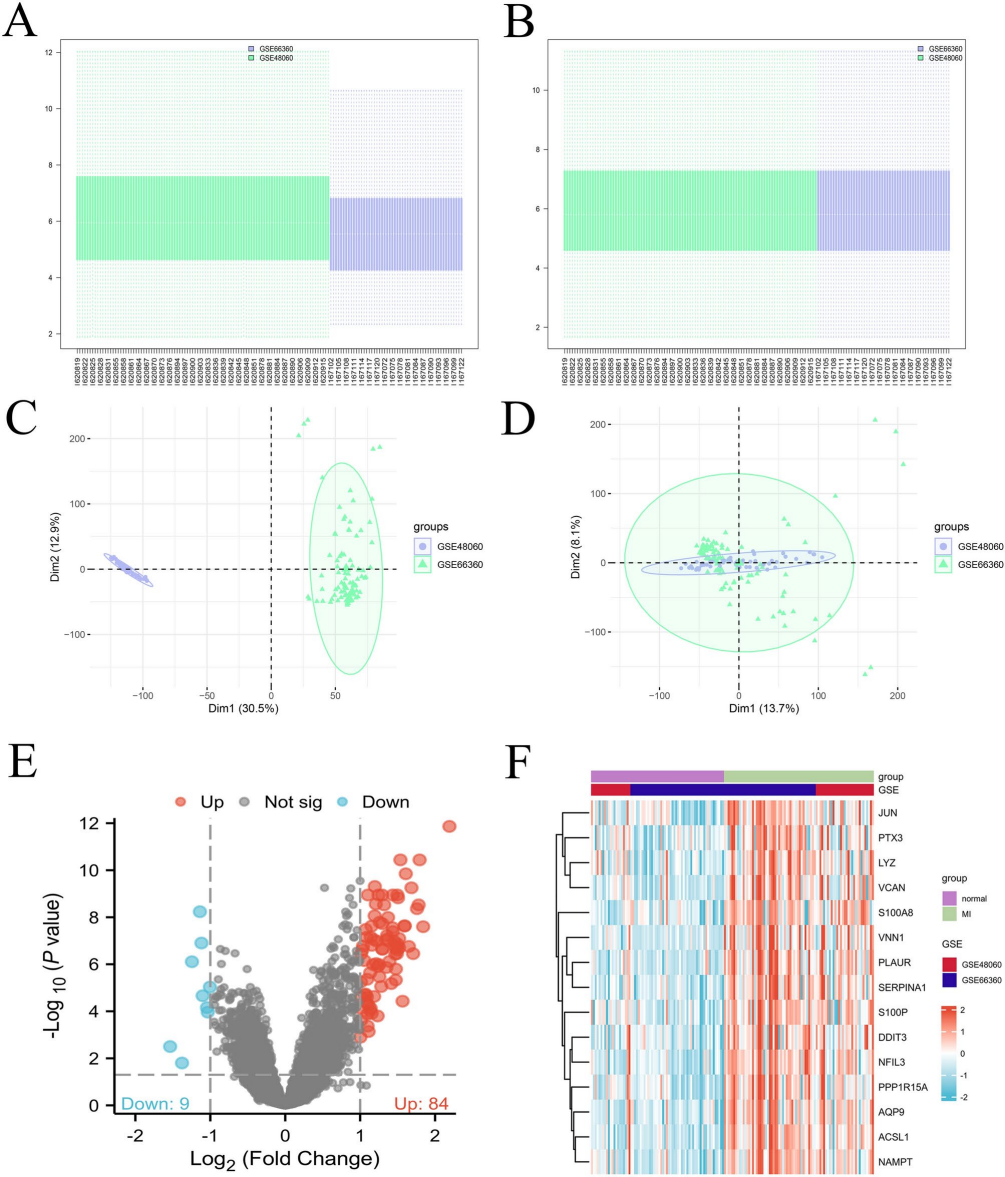
Identification the DEGs between healthy control and AMI patient samples. (**A, B**) Box plot shows pre- and post-normalization of two expression datasets (GSE48060 and GSE66360). (**C, D**) Principal Component Analysis (PCA) plots showing the chosen GEO datasets pre- and post- batch effect removal. (**E**) Volcano plot of the CRGs from combined database. Filtering criteria base on | log2FC |> 1 and adjusted *p* < 0.05 (Benjamini–Hochberg correction). (**F**) Heatmap of the CRGs from the combined database. Red indicates increased gene expression, while blue indicates decreased gene expression.

We obtained 71 healthy controls and 80 AMI patient samples data from two datasets (GSE48060 and GSE66360) from the GEO database. The expression data were normalized and visualized by box plots (Fig. 1A,B). The batch effect was corrected through the PCA algorithm (Fig. 1C,D). Then, through the Pearson correlation analysis, circadian rhythm genes were obtained using the “limma[3.52.2]” package in the R software (Version 4.2.1)^21^. To control for multiple comparisons, the Benjamini–Hochberg method was applied, and genes with adjusted *p*-values < 0.05 were considered statistically significant. Based on the criterion |log2FC |> 1 and *p* < 0.05 (Benjamini–Hochberg corrected), differential CRGs were identified and visualized by the volcano plot and heatmap (Fig. 1E,F).

### Functional analysis of the differential CRGs

**Fig. 2:**
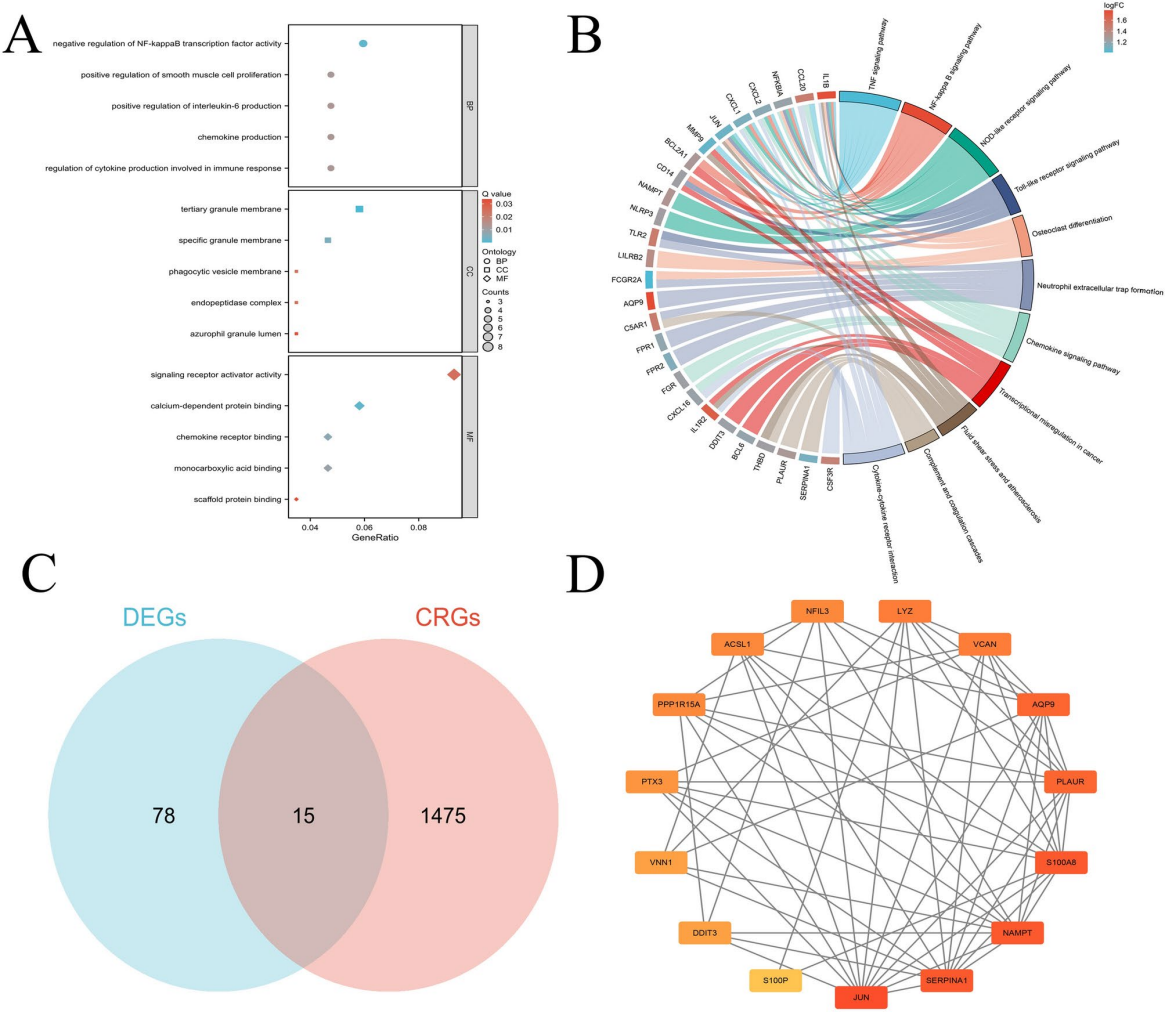
Functional enrichment analysis of differential CRGs. (**A**) GO analysis of differential CRGs. (**B**) Combined GO and KEGG analysis with logFC of differential genes. (**C**) Venn diagram showing the overlap between DEGs and CRGs, highlighting 15 genes common in two specimens. (**D**) PPI network of the 15 hub CRGs.

Functional enrichment analysis, with *p*-values adjusted using the Benjamini–Hochberg method, indicated that the differential CRGs were mainly involved in inflammatory and immune response biological process (BP) terms, including “negative regulation of NF-kappaB transcription factor activity”, “positive regulation of smooth muscle cell proliferation”, “positive regulation of interleukin-6 production”, “chemokine production”, and “regulation of cytokine production involved in immune response” (Fig. 2A). The cellular component (CC) analysis enriched in “tertiary granule membrane”, “specific granule membrane”, “phagocytic vesicle membrane”, “endopeptidase complex”, and “azurophil granule lumen” (Fig. 2A). The molecular function (MF) analysis enriched in “signaling receptor activator activity”, “calcium-dependent protein binding”, “chemokine receptor binding”, “monocarboxylic acid binding”, and “scaffold protein binding” (Fig. 2A). Our analysis revealed significant enrichment of DEGs in several key biological pathways, indicating potential mechanisms underlying the disease process. Notably, enriched pathways (adjusted *p* < 0.05, Benjamini–Hochberg corrected) included the complement and coagulation cascades, cytokine-cytokine receptor interaction, transcriptional misregulation in cancer, chemokine signaling pathway, neutrophil extracellular trap formation, NOD-like receptor signaling pathway, fluid shear stress and atherosclerosis, osteoclast differentiation, TNF signaling pathway and toll-like receptor signaling pathway (Fig. 2B). A total of 78 differentially expressed genes (DEGs, blue) and 1475 CRGs (red) were identified, with 15 genes overlapping between the two groups (Fig. 2C). Through network analysis using the Cytoscape software and the “Cytohubba” plugin, isolated nodes were excluded from the network, and 15 hub CRGs were identified, including JUN, PTX3, LYZ, VCAN, S100A8, VNN1, PLAUR, SERPINA1, S100P, DDIT3, NFIL3, PPP1R15A, AQP9, ACSL1, and NAMPT (Fig. 2D).

### Identification of hub CRGs

**Fig. 3:**
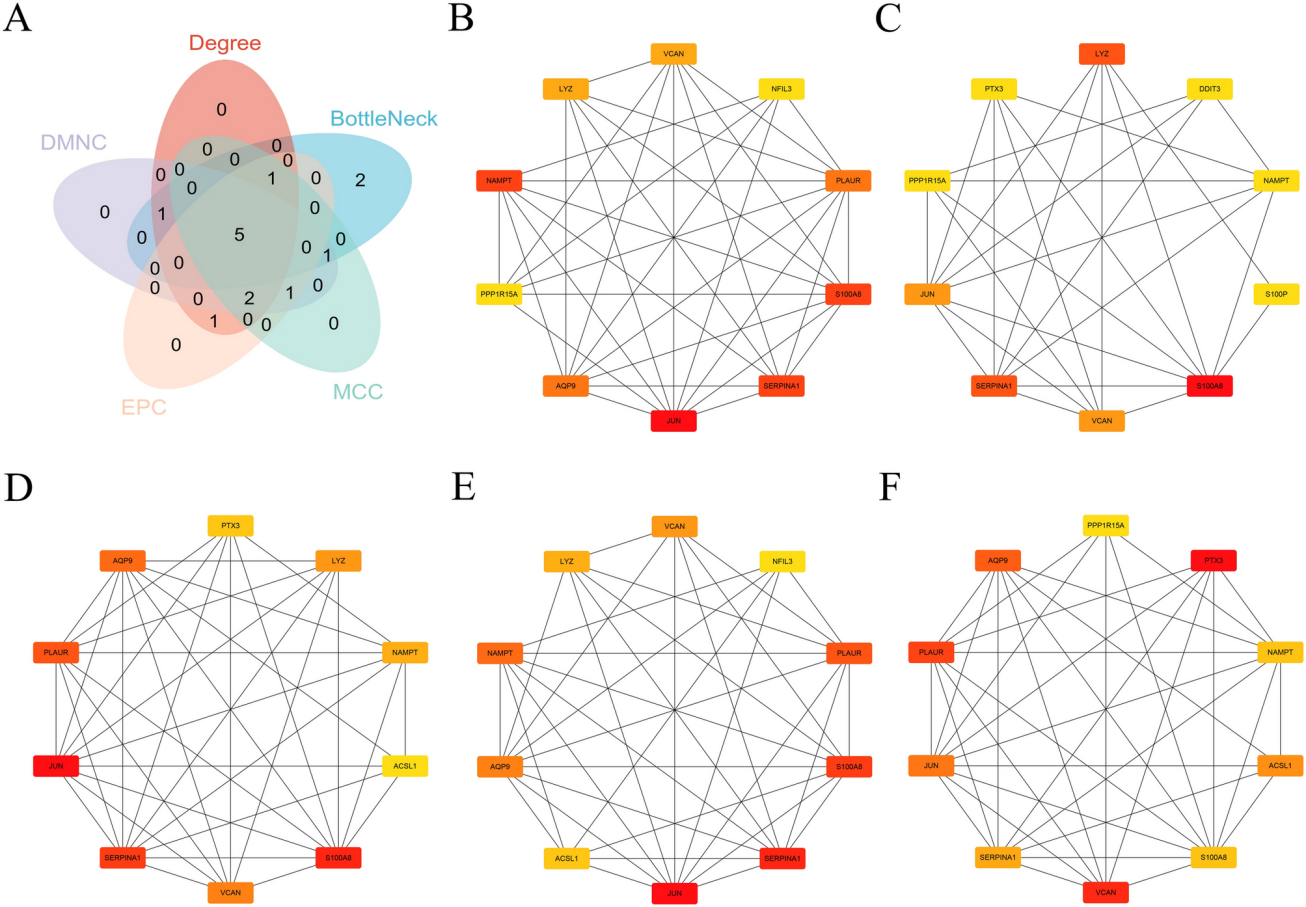
Determination of hub CRGs using multiple methods. (**A**) Venn diagram showing the overlap 5 methods in network including Degree, BottleNeck, MCC, EPC, and DMNC, highlighting 5 genes common in five methods. (**B**) Identification of top 10 hub CRGs with Degree method. (**C**) Identification of top 10 hub CRGs with BottleNeck method. (**D**) Identification of top 10 hub CRGs with MCC method. (**E**) Identification of top 10 hub CRGs with EPC method. (**F**) Identification of top 10 hub CRGs with DMNC method.

The Venn diagram illustrates the overlap in gene identification among five different methods: Degree, BottleNeck, DMNC, EPC and MCC (Fig. 3A). The central overlap indicates that 5 genes are identified by all five methods, namely S100A8, SERPINA1, VCAN, JUN, and NAMPT, suggesting their potential significance. The top 10 hub CRGs, including JUN, SERPINA1, NAMPT, S100A8, AQP9, PLAUR, VCAN, LYZ, PPP1R15A, and NFIL3, were identified by the Degree method (Fig. 3B). The BottleNeck method identified the top 10 hub CRGs as S100A8, SERPINA1, LYZ, VCAN, JUN, S100P, PTX3, DDIT3, NAMPT, and PPP1R15A (Fig. 3C). Using the MCC algorithm, the top 10 hub CRGs were determined to be JUN, S100A8, SERPINA1, PLAUR, AQP9, VCAN, LYZ, NAMPT, PTX3, and ACSL1 (Fig. 3D). The EPC method identified JUN, SERPINA1, S100A8, PLAUR, NAMPT, AQP9, VCAN, LYZ, ACSL1, and NFIL3 as the top 10 hub CRGs (Fig. 3E). According to the DMNC method, the top 10 hub CRGs were PTX3, VCAN, PLAUR, AQP9, JUN, ACSL1, SERPINA1, NAMPT, S100A8, and PPP1R15A (Fig. 3F).

### Identification of 5 feature genes for diagnosing AMI

**Fig. 4:**
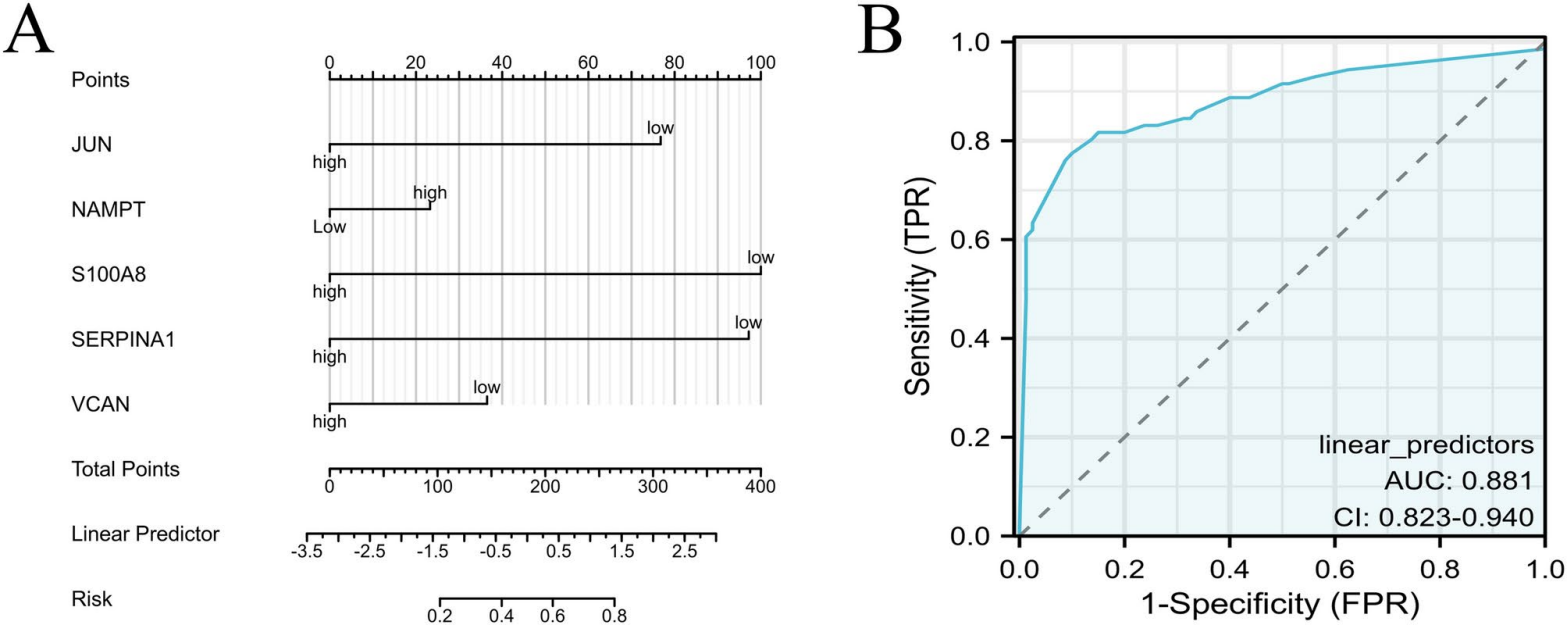
Diagnostic model construction. (**A**) Construction of a nomogram model with 5 feature genes. (**B**) ROC curve for evaluating 5 genes signature model’s diagnostic performance.

To identify diagnostic biomarkers for AMI, we utilized the 5 hub CRGs (S100A8, SERPINA1, VCAN, JUN, and NAMPT) as diagnostic feature genes to predict AMI and construct a nomogram (Fig. 4A). The diagnostic performance of the 5-gene signature model was evaluated using the ROC curve, with the training dataset yielding an AUC value of 0.881, indicating a promising predictive value for AMI (Fig. 4B). Nonetheless, the accuracy and reliability of this diagnostic model require further validation in future clinical trials.

### Identification of pivotal miRNAs and candidate drugs

**Fig. 5:**
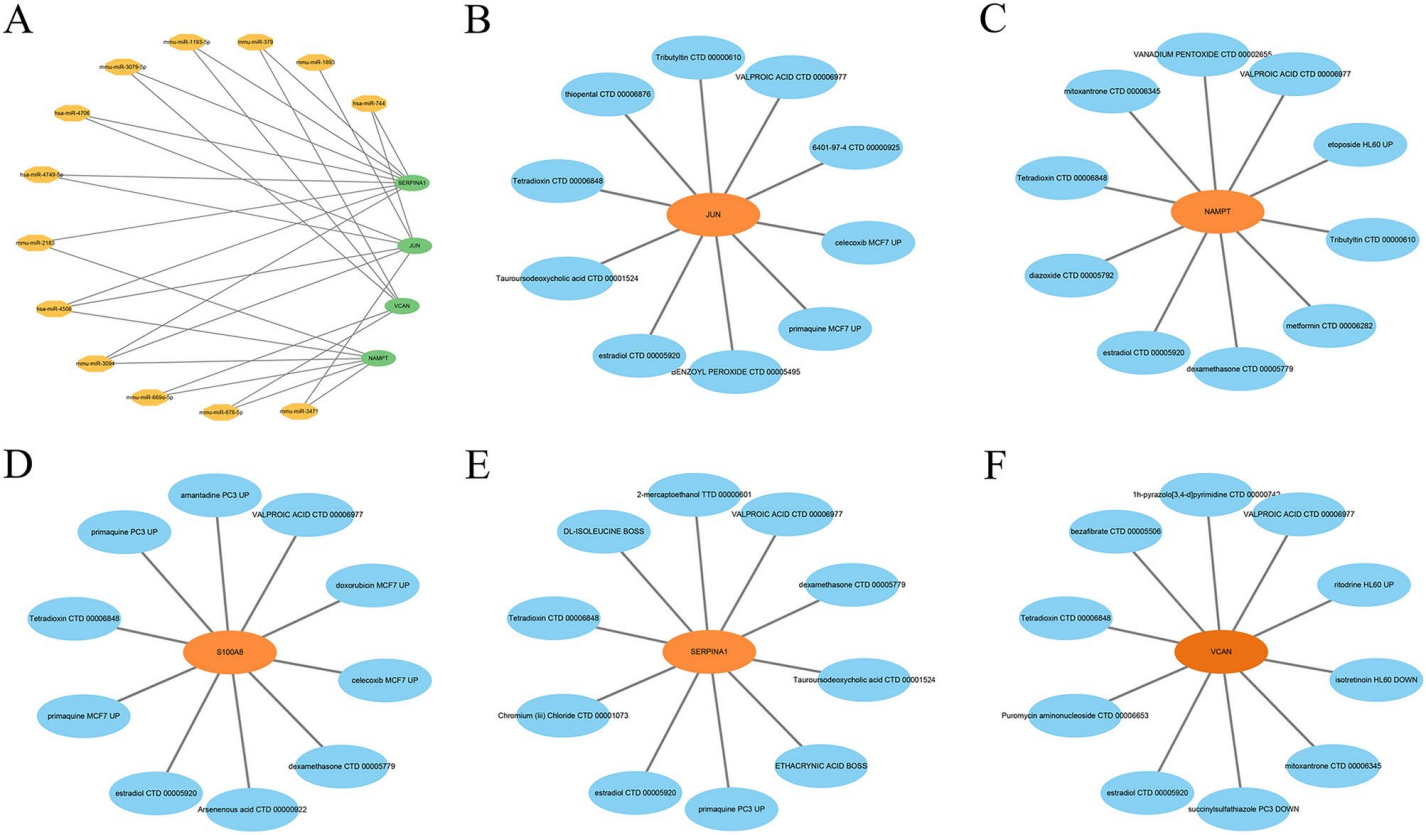
MiRNA-mRNA and drug-gene network construction. (**A**) 13 Pivotal miRNAs targeting 5 feature genes. (**B-F**) Ten candidate drug molecules targeting the 5 feature genes JUN, NAMPT, S100A8, SERPINA1, and VACN.

To identify the pivotal miRNAs and candidate drugs targeting the 5 feature genes, the data was collected from the Targetscan database and DSigDB database. Ultimately, a total of 13 miRNAs were screened with a set *p* < 0.05 (Fig. 5A). Notably, SERPINA1 could be regulated by all 9 miRNAs (hsa-miR-744, mmu-miR-379, mmu-miR-1193-5p, mmu-miR-3079-5p, hsa-miR-4706, hsa-miR-4749-5p, mmu-miR-2183, hsa-miR-4506, and mmu-miR-3094). JUN interacts with 7 miRNAs: hsa-miR-744, mmu-miR-1893, hsa-miR-4706, hsa-miR-4749-5p, hsa-miR-4506, mmu-miR-3094, and mmu-miR-3471). The VCAN interacted with 5 miRNAs: mmu-miR-379, mmu-miR-1193-5p, mmu-miR-3079-5p, mmu-miR-6690-5p, and mmu-miR-878-5p. NAMPT was demonstrated to interact with 6 miRNAs: mmu-miR-2183, hsa-miR-4506, mmu-miR-3094, mmu-miR-6690-5p, mmu-miR-878-5p, and mmu-miR-3471). We screened the top 10 drug molecules based on an adjusted *p* < 0.05 using the DSigDB database (Fig. 5B–5F). Among these, VALPROIC ACID CTD 00,006,977 and estradiol CTD 00,005,920 were associated with all five feature genes. Dexamethasone CTD 00,005,779 was linked to three feature genes, including S100A8, SERPINA1, and NAMPT. The remaining drug molecules also demonstrated interactions with the feature genes. These drug candidates offer promising avenues for further research and development in the treatment of AMI.

### Correlation matrix and infiltration analysis of immune cells and gene expression

**Fig. 6:**
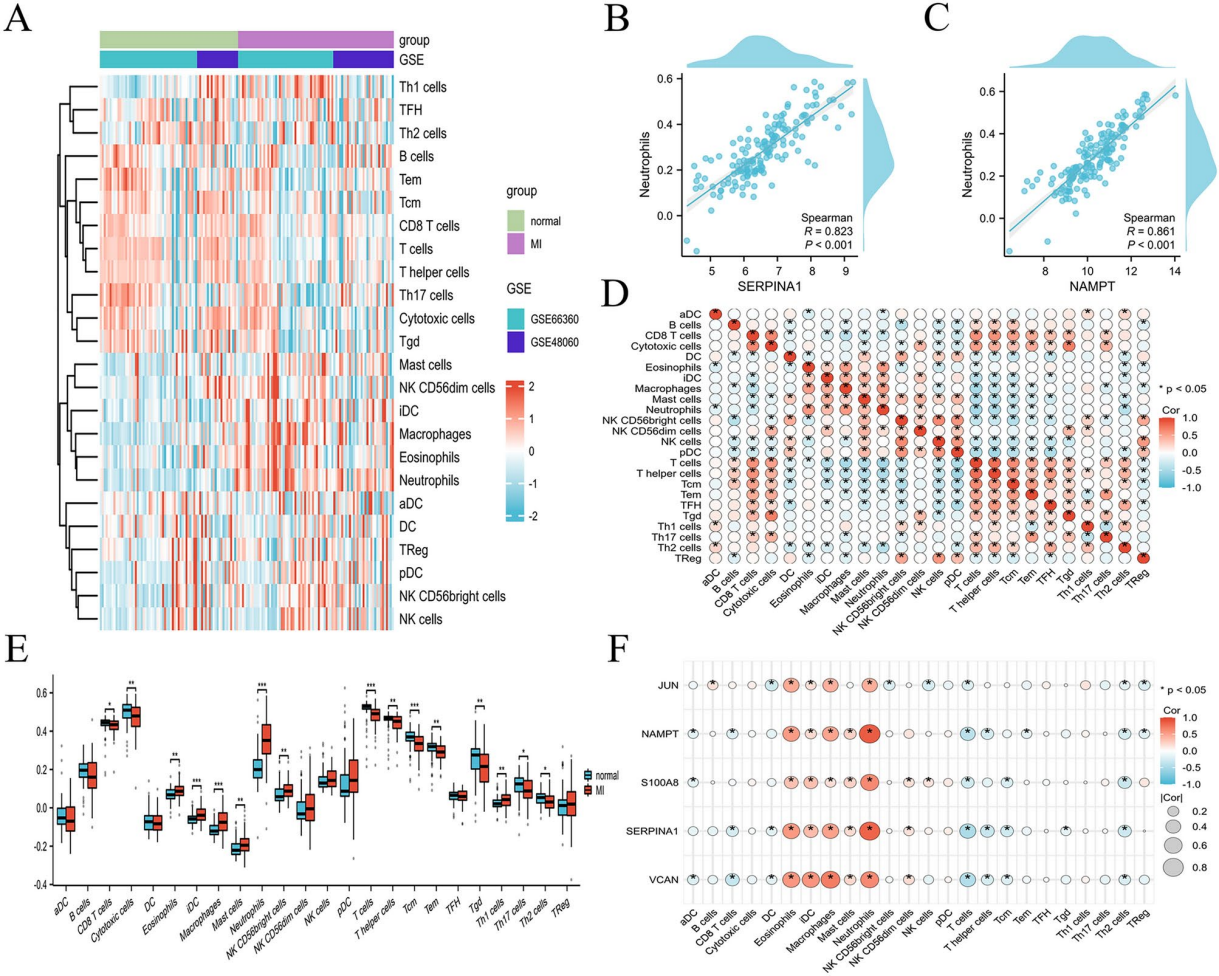
Correlation among hub CRDEGs with differentially infiltrated immune cells and functions in AMI patients and healthy controls. (**A**) Heatmap of differential immune cells and functions. (**B, C**) Scatter diagram of the correlation between neutrophils and SERPINA1 and NAMPT. (**D**) Correlation matrix of 24 immune cells. (**E**) The ssGSEA scores of 24 immune cells. (**F**) Heatmap of correlation among 5 hub CRDEGs with immune cells and functions (***p < 0.001, ** *p* < 0.01, * *p* < 0.05).

We further investigated the immune cell infiltration between AMI patients and healthy controls, the enrichment scores of distinct immune cell subpopulations and functions were assessed using ssGSEA. The results were visualized via the heatmap (Fig. 6A). AMI patients showed elevated levels of eosinophils, iDC, macrophages, mast cells, neutrophils, NK CD56 bright cells and Th1 cells, but decreased levels of CD8 T cells, cytotoxic cells, T cells, T helper cells, Tcm, Tem, Tgd, Th17 cells and Th2 cells (Fig. 6E). The correlation of 24 immune cells indicated that neutrophils were positively correlated with macrophages, eosinophils and mast cells, while neutrophils were negatively correlated with T cells and T helper cells. In addition, cytotoxic cells were positively connected with Tgd and CD8 T cells (Fig. 6D). These findings indicate that the immune cell infiltration patterns differ significantly between AMI patients and healthy individuals, potentially playing a crucial role in the pathophysiological processes of the disease. As shown in (Fig. 6F), the heatmap illustrates the correlation between the expression of 5 genes (S100A8, SERPINA1, VCAN, JUN, and NAMPT) and the abundance of different immune cell types. Notably, genes such as NAMPT and SERPINA1 show strong positive correlations with neutrophils, suggesting a potential role in the inflammatory response (Fig. 6B,C). Conversely, these genes tend to have negative correlations with T cells and Th2 cells, indicating a complex interaction between gene expression and immune regulation in the context of AMI.

### RT-qPCR validation of 5 CRGs in the AC16 hypoxia culture model

**Fig. 7:**
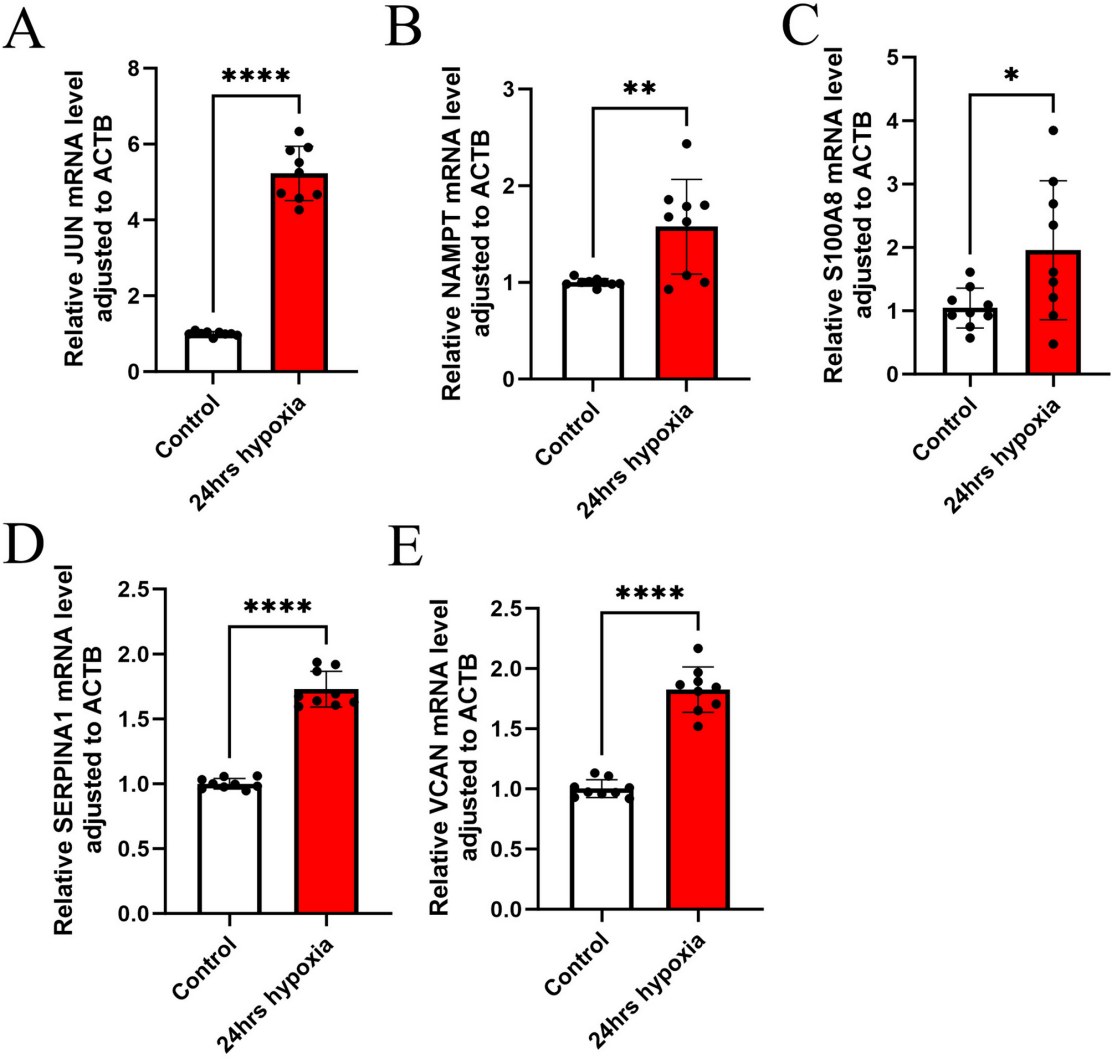
Genes expression in control and 24 h hypoxia in AC16 cells. Control and 24 h hypoxia mRNA levels of JUN (**A**) NAMPT (**B**) S100A8 (**C**) SERPINA1 (**D**) VCAN (**E**) in AC16 cells (****p < 0.0001, ** *p* < 0.01, * *p* < 0.05).

RT-qPCR was performed to quantify the expression of the five CRGs in AC16 cells exposed to 24 h of hypoxia, aiming to validate the bioinformatics analysis results. As predicted, the results showed that the five key genes (S100A8, SERPINA1, VCAN, JUN, and NAMPT) were highly expressed in AC16 under hypoxic conditions compared to normoxic conditions (Fig. 7A–E). These findings indicate the potential of these genes as promising diagnostic targets.

## Discussion

Circadian rhythm-related genes (CRGs) play a crucial role in coordinating the regulation of circadian rhythms, the body’s endogenous timing system. Emerging evidence has well established the impact of circadian rhythms on cardiovascular function and myocardial ischemia injury, strongly supporting CRGs as candidate diagnostic biomarkers^34,35^. Simultaneously, a study reveals a potentially complex relationship between CRGs and immune infiltration in heart failure^19^. In the present study, we used bioinformatics tools to investigate the role of CRGs in AMI as well as their interaction with immune infiltration, attempting to identify more precise AMI diagnostic markers and treatment targets.

We systematically screened 15 differential CRGs in the peripheral blood of 71 healthy controls and 80 AMI patients. Functional enrichment analysis revealed that the differential CRGs were enriched in the regulation of cytokine production involved in immune response and the chemokine production. Cytokines and chemokines are essential mediators that orchestrate the inflammatory response in atherosclerosis procession, such as monocyte/lymphocyte recruitment, regulating plaque stability, rupture and thrombus formation^36–38^. During acute myocardial infarction, the balance of pro-inflammatory and anti-inflammatory cytokines can influence the recruitment immune cells to the site of injury, facilitate repair processes and subsequent cardiac remodeling^39,40^. These supported that CRGs might play an important role in the initiation and subsequent AMI via inflammatory responses. Then, we constructed the PPI network and screened 15 hub CRGs, namely JUN, PTX3, LYZ, VCAN, S100A8, VNN1, PLAUR, SERPINA1, S100P, DDIT3, NFIL3, PPP1R15A, AQP9, ACSL1, and NAMPT, all of which are potential candidate biomarkers closely related to AMI.

AMI patients showed elevated levels of neutrophils, positively correlating with macrophages, implying the clearance of necrotic cardiomyocytes and repair processes^41^. An in vivo study showed that neutrophils induce macrophages towards a reparative phenotype via neutrophil gelatinase-associated lipocalin in the MI^42^. The resolution of inflammation is marked by the efflux of macrophages via the lymphatic system^41^. Our results showed that mast cells and eosinophils were increased in AMI patients, positively relating to neutrophils. To date, the impact of mast cells on infarcted myocardial tissue remains a subject of ongoing debate. Nonetheless, mast cells are implicated in protective mechanisms after MI, including promoting angiogenesis, regulating cardiomyocyte contractility, enhancing hypoxia resistance and facilitating the conversion of fibroblasts to myofibroblasts^43^. In addition, eosinophils have shown potential as biomarkers for AMI and play a role in tissue repair processes^44–46^. In the present study, we demonstrated that the immune function of neutrophil was elevated in the AMI group. In the follow-up analysis, we identified the 5 hub CRGs (S100A8, SERPINA1, VCAN, JUN, and NAMPT) most associated with immune infiltration as diagnostic feature genes. The expression levels of all 5 feature genes were strongly positively linked with the neutrophils. These suggest that the 5 feature genes participate in the immune and inflammatory responses of AMI, through neutrophil signaling pathways. In AMI patients, the S100A8 levels positively were correlated with neutrophil counts^47^. Furthermore, S100A8 is associated with cardiac rupture, serving as a robust predictor and a potentially causal mediator^48,49^. Consistent with our result, a weighted gene co-expression network analysis research showed that the SERPINA1 was identified and validated for the predictive value in identifying future heart failure after AMI^50^. In our study, the SERPINA1 was highly expressed under the 24 h hypoxia culture, with validation achieved through RT-qPCR. VCAN, as one of the ten strongly interlinked hub genes, was identified in the remodeling of non-infarcted myocardium following acute myocardial infarction^51^. Similarly, our study also confirmed the VCAN high expression in response to hypoxia. Studies showed that JUN involved in the MI process^52,53^. It has been documented that inhibition of NAMPT may attenuate tissue damage mediated by neutrophilic inflammation and oxidative stress during the initial stages of re-perfusion following myocardial infarction^54,55^. These findings indicate that these 5 central CRGs may represent promising diagnostic and therapeutic targets for AMI.

We predicted the miRNAs and candidate drugs that regulate the five CRGs. Hsa-miR-4506 was identified as a regulator of three key genes (SERPINA1, JUN, and NAMPT) and has been recognized as a highly predictive miRNA for colon and rectal cancer^56^. Hsa-miR-744, which regulates SERPINA1 and JUN, has been found to be elevated in various cancers, with significant implications for prognosis^57–59^. Studies have shown that up-regulation of mmu-miR-379 occurs in both the plasma of Crohn’s disease patients and the development of obesity, the latter being associated with an increased risk of cardiovascular disease^60,61^. Hsa-miR-4706 was found to be elevated in people blood samples with head and neck cancer (HNC), suggesting its potential as a predictive biomarker for HNC^62^. Additionally, our study is the first to report the involvement of mmu-miR-3471, mmu-miR-1893, mmu-miR-1193-5p, mmu-miR-3079-5p, hsa-miR-4749-5p, mmu-miR-2183, and mmu-miR-3094 in relation to CRGs in AMI. These miRNAs may serve as independent predictors of AMI, however, their specific mechanisms of action in AMI require further investigation.

Among all candidate drugs, VALPROIC ACID CTD 00,006,977 and estradiol CTD 00,005,920 ranked in the top two with targeting all 5 feature CRGs. Dexamethasone CTD 00,005,779 was linked to three feature genes, including S100A8, SERPINA1, and NAMPT. The VALPROIC ACID CTD 00,006,977, the drug identified in our screening, serves as a reverse agonist of the retinoic acid-related orphan receptor α (RORα)^63^. RORα is involved in the regulation of inflammatory macrophages under pathological conditions, such as myocardial infarction^64^. These suggest that VALPROIC ACID CTD 00,006,977 might affect the process of AMI by impacting genes involved in CRGs. The potential effectiveness of other proposed drugs is also being considered, and these drugs may warrant further validation through chemical experiments.

In summary, this study used bioinformatics methods to analyze the transcriptional expression characteristics of AMI and screened five biomarkers (S100A8, SERPINA1, VCAN, JUN, and NAMPT) related to AMI. Drug database enrichment found that these five CRGs may be the drug targets of AMI and VALPROIC ACID CTD 00,006,977 and estradiol CTD 00,005,920 may be a potential targeted therapeutic drug. Hsa-miR-4506 plays an important role in regulating CRGs in AMI. Future research will prioritize large-scale cohorts encompassing diverse ethnic groups to further validate the generalizability of our findings across populations and address potential limitations of the present study. While our discoveries are notable, the study is constrained by limitations such as only in vitro validation and the absence of clinical specimens. Future research will focus on obtaining patient samples through collaborations with clinical teams to overcome the limitation of relying solely on in vitro cell experiments and to further strengthen the translational relevance of the identified biomarkers. Additionally, further experimentation is necessary to demonstrate the effects and underlying mechanisms of other miRNAs. By addressing these limitations, future research can provide novel insights into the diagnosis and management of AMI.

## Conclusions

This study highlights the critical interplay between circadian rhythm-related genes and immune infiltration in the pathogenesis of acute myocardial infarction (AMI). Our findings underscore the potential of circadian rhythm-related biomarkers to enhance the early diagnosis of AMI, bridging a gap in the current diagnostic landscape. The identification of five key immune-related circadian rhythm genes and their validation through bioinformatics and experimental models lay the groundwork for future translational research. These insights provide a promising foundation for the development of more precise diagnostic tools and therapeutic strategies targeting AMI.

## Data Availability

Data analysed was obtained from Gene Expression Omnibus database under accession number GSE48060 and GSE66360, are available at the following URL: https://www.ncbi.nlm.nih.gov/geo/query/acc.cgi?acc=GSE48060, https://www.ncbi.nlm.nih.gov/geo/query/acc.cgi?acc=GSE66360; The PPI network of the differential genes were constructed via string database, are available at the following URL: https://www.string-db.org/; The candidate drugs were obtained from DSigDB database, are available at the following URL: https://dsigdb.tanlab.org/DSigDBv1.0/geneSearch.html. Pivotal miRNAs were obtained from miRDB database, are available at the following URL: http://mirdb.org/miRDB/

## References

[1] Oliveira, G. B. F., Avezum, A. & Roever, L. Cardiovascular disease burden: Evolving knowledge of risk factors in myocardial infarction and stroke through population-based research and perspectives in global prevention. *Front. Cardiovasc. Med.* **2**, 32 (2015). 26664903 10.3389/fcvm.2015.00032 PMC4671335

[2] Rakic, M. et al. Possible role of circulating endothelial cells in patients after acute myocardial infarction. *Med. Hypotheses* **117**, 42–46 (2018). 30077195 10.1016/j.mehy.2018.06.005

[3] Rezaei, Z. & Ranjbar, B. Ultra-sensitive, rapid gold nanoparticle-quantum dot plexcitonic self-assembled aptamer-based nanobiosensor for the detection of human cardiac troponin I. *Eng. Life Sci.* **17**, 165–174 (2017). 32624764 10.1002/elsc.201500188 PMC6999242

[4] de Winter, R. J., Koster, R. W., Sturk, A. & Sanders, G. T. Value of myoglobin, troponin T, and CK-MBmass in ruling out an acute myocardial infarction in the emergency room. *Circulation* **92**, 3401–3407 (1995). 8521560 10.1161/01.cir.92.12.3401

[5] Mair, J. et al. Early diagnosis of acute myocardial infarction by a newly developed rapid immunoturbidimetric assay for myoglobin. *British Heart J.* **68**, 462–468 (1992). 10.1136/hrt.68.11.462 PMC1025188 1467029

[6] Bertrand, M. E. et al. Management of acute coronary syndromes: Acute coronary syndromes without persistent ST segment elevation; recommendations of the Task Force of the European Society of Cardiology. *Eur. Heart J.* **21**, 1406–1432 (2000). 10952834 10.1053/euhj.2000.2301

[7] Ang, E. et al. Cardiac troponin I and T in checkpoint inhibitor-associated myositis and myocarditis. *J. Immunother.* **44**, 162–163 (2021). 33416262 10.1097/CJI.0000000000000356

[8] Mahajan, N., Mehta, Y., Rose, M., Shani, J. & Lichstein, E. Elevated troponin level is not synonymous with myocardial infarction. *Int. J. Cardiol.* **111**, 442–449 (2006). 16290105 10.1016/j.ijcard.2005.08.029

[9] Lecour, S. et al. Circadian rhythms in ischaemic heart disease: key aspects for preclinical and translational research: Position paper of the ESC working group on cellular biology of the heart. *Cardiovasc. Res.* **118**, 2566–2581 (2022). 34505881 10.1093/cvr/cvab293

[10] Sehgal, A., Price, J. L., Man, B. & Young, M. W. Loss of circadian behavioral rhythms and per RNA oscillations in the Drosophila mutant timeless. *Science (New York, NY)* **263**, 1603–1606 (1994). 10.1126/science.8128246 8128246

[11] Rabinovich-Nikitin, I., Kirshenbaum, E. & Kirshenbaum, L. A. Autophagy, clock genes, and cardiovascular disease. *Canad. J. Cardiol.* **39**, 1772–1780 (2023). 37652255 10.1016/j.cjca.2023.08.022

[12] Delisle, B. P. et al. Circadian regulation of cardiac arrhythmias and electrophysiology. *Circul. Res.* **134**, 659–674 (2024). 10.1161/CIRCRESAHA.123.323513 PMC11177776 38484028

[13] Sartor, F. et al. Circadian clock and hypoxia. *Circul. Res.* **134**, 618–634 (2024). 10.1161/CIRCRESAHA.124.323518 38484033

[14] Man, A. W. C., Li, H. G. & Xia, N. Circadian rhythm: Potential therapeutic target for atherosclerosis and thrombosis. *Int. J. Mol. Sci.* **22**, 676 (2021). 33445491 10.3390/ijms22020676 PMC7827891

[15] Rijo-Ferreira, F. & Takahashi, J. S. Genomics of circadian rhythms in health and disease. *Genome Med.* **11**, 82 (2019). 31847894 10.1186/s13073-019-0704-0 PMC6916512

[16] Sakakura, K. et al. Pathophysiology of atherosclerosis plaque progression. *Heart Lung Circul.* **22**, 399–411 (2013). 10.1016/j.hlc.2013.03.001 23541627

[17] Prabhu, S. D. & Frangogiannis, N. G. The biological basis for cardiac repair after myocardial infarction from inflammation to fibrosis. *Circul. Res.* **119**, 91–112 (2016). 10.1161/CIRCRESAHA.116.303577 PMC4922528 27340270

[18] Mann, D. L. Innate immunity and the failing heart the cytokine hypothesis revisited. *Circul. Res.* **116**, 1254–1268 (2015). 10.1161/CIRCRESAHA.116.302317 PMC4380242 25814686

[19] Wang, X. F., Rao, J., Zhang, L., Liu, X. W. & Zhang, Y. F. Identification of circadian rhythm-related gene classification patterns and immune infiltration analysis in heart failure based on machine learning. *Heliyon* **10**, (2024). 10.1016/j.heliyon.2024.e27049 PMC10950509 38509983

[20] Chi, H. et al. Circadian rhythm-related genes index: A predictor for HNSCC prognosis, immunotherapy efficacy, and chemosensitivity. *Front. Immunol.* **14**, 1091218 (2023). 36969232 10.3389/fimmu.2023.1091218 PMC10036372

[21] Smyth, G. K. Limma: Linear models for microarray data. In Bioinformatics and computational biology solutions using R and Bioconductor (2013).

[22] Leek, J. T., Johnson, W. E., Parker, H. S., Jaffe, A. E. & Storey, J. D. The <tt>sva</tt> package for removing batch effects and other unwanted variation in high-throughput experiments. *Bioinformatics* **28**, 882–883 (2012). 22257669 10.1093/bioinformatics/bts034 PMC3307112

[23] Wu, Y. M. et al. Development and validation of a novel circadian rhythm-related signature to predict the prognosis of the patients with hepatocellular carcinoma. *Biomed. Res. Int.* **2022**, 4263261 (2022). 35993051 10.1155/2022/4263261 PMC9391189

[24] Zhou, R. R. et al. A circadian rhythm-related gene signature associated with tumor immunity, cisplatin efficacy, and prognosis in bladder cancer. *Aging-Us* **13**, 25153–25179 (2021). 10.18632/aging.203733 PMC8714136 34862329

[25] Yu, G. C., Wang, L. G., Han, Y. Y. & He, Q. Y. clusterProfiler: An R package for comparing biological themes among gene clusters. *Omics-a J. Integr. Biol.* **16**, 284–287 (2012). 10.1089/omi.2011.0118 PMC3339379 22455463

[26] Kanehisa, M. & Goto, S. KEGG: Kyoto encyclopedia of genes and genomes. *Nucleic Acids Res.* **28**, 27–30 (2000). 10592173 10.1093/nar/28.1.27 PMC102409

[27] Kanehisa, M. Toward understanding the origin and evolution of cellular organisms. *Protein Sci.* **28**, 1947–1951 (2019). 31441146 10.1002/pro.3715 PMC6798127

[28] Kanehisa, M., Furumichi, M., Sato, Y., Kawashima, M. & Ishiguro-Watanabe, M. KEGG for taxonomy-based analysis of pathways and genomes. *Nucleic Acids Res.* **51**, D587–D592 (2023). 36300620 10.1093/nar/gkac963 PMC9825424

[29] Park, T. J. et al. Quantitative proteomic analyses reveal that GPX4 downregulation during myocardial infarction contributes to ferroptosis in cardiomyocytes. *Cell Death Dis.* **10**, 835 (2019). 31685805 10.1038/s41419-019-2061-8 PMC6828761

[30] Dailey, A. L. Metabolomic bioinformatic analysis. *Methods Mol. Biol. (Clifton, NJ)* **1606**, 341–352 (2017). 10.1007/978-1-4939-6990-6_22 28502011

[31] Hu, K. Become competent within one day in generating boxplots and violin plots for a novice without prior R experience. *Methods Protocols* **3**, 64 (2020). 32977580 10.3390/mps3040064 PMC7712237

[32] Hänzelmann, S., Castelo, R. & Guinney, J. GSVA: Gene set variation analysis for microarray and RNA-Seq data. *Bmc Bioinform.* **14**, 1–15 (2013). 10.1186/1471-2105-14-7 PMC3618321 23323831

[33] Bindea, G. et al. Spatiotemporal dynamics of intratumoral immune cells reveal the immune landscape in human cancer. *Immunity* **39**, 782–795 (2013). 24138885 10.1016/j.immuni.2013.10.003

[34] Eckle, T. et al. Circadian influences on myocardial ischemia-reperfusion injury and heart failure. *Circul. Res.* **134**, 675–694 (2024). 10.1161/CIRCRESAHA.123.323522 PMC10947118 38484024

[35] Lin, J. Y. et al. Circadian rhythms in cardiovascular function: Implications for cardiac diseases and therapeutic opportunities. *Med. Sci. Monitor* **29**, 1837–1844 (2023). 10.12659/MSM.942215 PMC10675984 37986555

[36] Ma, J. et al. The roles of B cells in cardiovascular diseases. *Mol. Immunol.* **171**, 36–46 (2024). 38763105 10.1016/j.molimm.2024.05.002

[37] Suryan, V. & Chandra, N. C. Cholesterol and cytokines: Molecular links to atherosclerosis and carcinogenesis. *Cell Biochem. Biophys.* **82**, 1837–1844 (2024). 38943010 10.1007/s12013-024-01383-w

[38] Tedgui, A. & Mallat, Z. Cytokines in atherosclerosis: Pathogenic and regulatory pathways. *Physiol. Rev.* **86**, 515–581 (2006). 16601268 10.1152/physrev.00024.2005

[39] Ong, S. B. et al. Inflammation following acute myocardial infarction: Multiple players, dynamic roles, and novel therapeutic opportunities. *Pharmacol. Therap.* **186**, 73–87 (2018). 29330085 10.1016/j.pharmthera.2018.01.001 PMC5981007

[40] Frangogiannis, N. G. The immune system and cardiac repair. *Pharmacol. Res.* **58**, 88–111 (2008). 18620057 10.1016/j.phrs.2008.06.007 PMC2642482

[41] Serhan, C. N. & Savill, J. Resolution of inflammation: The beginning programs the end. *Nat. Immunol.* **6**, 1191–1197 (2005). 16369558 10.1038/ni1276

[42] Horckmans, M. et al. Neutrophils orchestrate post-myocardial infarction healing by polarizing macrophages towards a reparative phenotype. *Eur. Heart J.* **38**, 187–197 (2017). 28158426 10.1093/eurheartj/ehw002

[43] Xu, J. Y., Xiong, Y. Y., Lu, X. T. & Yang, Y. J. Regulation of type 2 immunity in myocardial infarction. *Front. Immunol.* **10**, 62 (2019). 30761134 10.3389/fimmu.2019.00062 PMC6362944

[44] Konishi, T. et al. Prognostic value of eosinophil to leukocyte ratio in patients with ST-elevation myocardial infarction undergoing primary percutaneous coronary intervention. *J. Atheroscler. Thromb.* **24**, 827–840 (2017). 27904028 10.5551/jat.37937 PMC5556190

[45] Niccoli, G. et al. Pre-intervention eosinophil cationic protein serum levels predict clinical outcomes following implantation of drug-eluting stents. *Eur. Heart J.* **30**, 1340–1347 (2009). 19383735 10.1093/eurheartj/ehp120

[46] Heredia, J. E. et al. Type 2 innate signals stimulate fibro/adipogenic progenitors to facilitate muscle regeneration. *Cell* **153**, 376–388 (2013). 23582327 10.1016/j.cell.2013.02.053 PMC3663598

[47] Sreejit, G. et al. Neutrophil-derived S100A8/A9 amplify granulopoiesis after myocardial infarction. *Circulation* **141**, 1080–1094 (2020). 31941367 10.1161/CIRCULATIONAHA.119.043833 PMC7122461

[48] Shi, S. & Yi, J. L. S100A8/A9 promotes MMP-9 expression in the fibroblasts from cardiac rupture after myocardial infarction by inducing macrophages secreting TNFα. *Eur. Rev. Med. Pharmacol. Sci.* **22**, 3925–3935 (2018). 29949169 10.26355/eurrev_201806_15278

[49] Ma, J. et al. S100A8/A9 as a prognostic biomarker with causal effects for post-acute myocardial infarction heart failure. *Nat. Commun.* **15**, 2701 (2024). 38538601 10.1038/s41467-024-46973-7 PMC10973499

[50] Niu, X. et al. Weighted gene co-expression network analysis identifies critical genes in the development of heart failure after acute myocardial infarction. *Front. Genet.* **10**, 1214 (2019). 31850068 10.3389/fgene.2019.01214 PMC6889910

[51] Wang, L., Zhang, Y., Yu, M. & Yuan, W. Identification of hub genes in the remodeling of non-infarcted myocardium following acute myocardial infarction. *J. Cardiovasc. Dev. Dis.* **9**, 409 (2022). 36547406 10.3390/jcdd9120409 PMC9788553

[52] Wu, Y. et al. CAV1 protein encapsulated in mouse BMSC-derived extracellular vesicles alleviates myocardial fibrosis following myocardial infarction by blocking the TGF-β1/SMAD2/c-JUN Axis. *J. Cardiovasc. Transl. Res.* **17**(3), 523–539 (2023). 38092988 10.1007/s12265-023-10472-9

[53] Reiss, K. et al. ANG II receptors, c-myc, and c-jun in myocytes after myocardial infarction and ventricular failure. *Am. J. Physiol.* **264**, H760-769 (1993). 8456979 10.1152/ajpheart.1993.264.3.H760

[54] Montecucco, F. et al. Inhibition of nicotinamide phosphoribosyltransferase reduces neutrophil-mediated injury in myocardial infarction. *Antioxidants Redox Signal.* **18**, 630–641 (2013). 10.1089/ars.2011.4487 PMC3549207 22452634

[55] Wang, S. & Cao, N. Uncovering potential differentially expressed miRNAs and targeted mRNAs in myocardial infarction based on integrating analysis. *Mol. Med. Rep.* **22**, 4383–4395 (2020). 33000230 10.3892/mmr.2020.11517 PMC7533449

[56] Pellatt, D. F. et al. Expression profiles of miRNA subsets distinguish human colorectal carcinoma and normal colonic mucosa. *Clin. Transl. Gastroenterol.* **7**, e152 (2016). 26963002 10.1038/ctg.2016.11 PMC4822091

[57] Ab Mutalib, N.-S. et al. Differential microRNA expression and identification of putative miRNA targets and pathways in head and neck cancers. *Int. J. Mol. Med.* **28**, 327–336 (2011). 21637912 10.3892/ijmm.2011.714

[58] Phuah, N. H. et al. Alterations of MicroRNA expression patterns in human cervical carcinoma cells (Ca Ski) toward 1′<i>S</i>-1′-acetoxychavicol acetate and cisplatin. *Reprod. Sci.* **20**, 567–578 (2013). 23012319 10.1177/1933719112459220 PMC3713538

[59] Qiao, Z. et al. Hsa-miR-557 inhibits osteosarcoma growth through targeting KRAS. *Front. Genet.* **12**, 789823 (2022). 35087570 10.3389/fgene.2021.789823 PMC8787190

[60] Jensen, M. D. et al. Circulating microRNAs as biomarkers of adult Crohn’s disease. *Eur. J. Gastroenterol. Hepatol.* **27**, 1038–1044 (2015). 26230660 10.1097/MEG.0000000000000430

[61] Chartoumpekis, D. V. et al. Differential expression of MicroRNAs in adipose tissue after long-term high-fat diet-induced obesity in mice. *Plos One* **7**, e34872 (2012). 22496873 10.1371/journal.pone.0034872 PMC3319598

[62] Torso, N. D. G. et al. miR-6805-5p as a biomarker of cisplatin-induced nephrotoxicity in patients with head and neck cancer. *Front. Pharmacol.* **14**, 893301 (2023). 10.3389/fphar.2023.1275238 PMC10713822 38089043

[63] Liu, G., He, G., Zhang, J., Zhang, Z. & Wang, L. Identification of SCRG1 as a potential therapeutic target for human synovial inflammation. *Front. Immunol.* **13**, 893301 (2022). 35720295 10.3389/fimmu.2022.893301 PMC9204521

[64] Cho, D. I. *et al.* Antiinflammatory activity of ANGPTL4 facilitates macrophage polarization to induce cardiac repair. *Jci Insight* **4**,(2019). 10.1172/jci.insight.125437 PMC6777833 31434807

